# Effects of Heat Stress during Anthesis and Grain Filling Stages on Some Physiological and Agronomic Traits in Diverse Wheat Genotypes

**DOI:** 10.3390/plants13152083

**Published:** 2024-07-27

**Authors:** Milan Mirosavljević, Sanja Mikić, Vesna Župunski, Lamis Abdelhakim, Dragana Trkulja, Rong Zhou, Ankica Kondić Špika, Carl-Otto Ottosen

**Affiliations:** 1 Institute of Field and Vegetable Crops, 21000 Novi Sad, Serbia; milan.mirosavljevic@ifvcns.ns.ac.rs (M.M.); sanja.mikic@ifvcns.ns.ac.rs (S.M.); vesna.zupunski@ifvcns.ns.ac.rs (V.Ž.); dragana.trkulja@ifvcns.ns.ac.rs (D.T.); 2 Department of Food Science, Aarhus University, 8200 Aarhus, Denmark; lamisabdelhakim@gmail.com (L.A.); rong.zhou@food.au.dk (R.Z.); coo@food.au.dk (C.-O.O.)

**Keywords:** chlorophyll fluorescence, flowering, grain filling, high temperature, leaf temperature, *Triticum aestivum* L.

## Abstract

Heat stress represents a significant environmental challenge that adversely impacts the growth, physiology, and productivity of wheat. In order to determine the response to high temperatures of the wheat varieties developed mostly in the Pannonian environmental zone, as well as varietal differences, we subjected seven varieties from Serbia, one from Australia, and one from the UK to thermal stress during anthesis and mid-grain filling and combined stress during both of these periods. The changes in chlorophyll fluorescence and index, leaf temperature, and main agronomic traits of nine winter wheat varieties were investigated under high temperatures. Heat stress negatively affected leaf temperature, chlorophyll fluorescence, and the chlorophyll index during different growth stages. Compared to the control, stress at anthesis, mid-grain filling, and combined stress resulted in yield reductions of 32%, 46%, and 59%, respectively. Single treatment at anthesis had a more severe effect on the number of grains per plant, causing a 38% reduction compared to the control. Moreover, single treatment during mid-grain filling resulted in the greatest decline in grain weight, with a 29% reduction compared to the control. There was a significant varietal variation in heat tolerance, highlighting Avangarda and NS 40s as the most tolerant varieties that should be included in regular breeding programs as valuable sources of heat tolerance. Understanding the genetic and physiological mechanisms of heat tolerance in these promising varieties should be the primary focus of future research and help develop targeted breeding strategies and agronomic practices to mitigate the adverse effects of heat stress on wheat production.

## 1. Introduction

Wheat is the most produced and processed cereal crop in Europe, contributing to more than one-third of global wheat production in 2022 [1]. Winter wheat is a major winter crop in Serbia, and it plays a crucial role in the country’s agricultural sector, contributing significantly to both domestic consumption and export markets. Wheat is cultivated on approximately 600,000 hectares, with average yields around 5.0 t ha^−1^. Although wheat yields in Europe are higher than the average global yield, they are often limited by different unfavorable environmental factors that vary across the continent. The conditions are rather favorable for wheat cultivation in Western Europe, resulting in average grain yields over 8 t ha^−1^ in Belgium, Ireland, the Netherlands, and the UK in recent years [1]. Contrary to Western Europe, the countries located in the Pannonian environmental zone are more susceptible to drought and thermal stress, often leading to significantly lower wheat grain yields [2]. Farmers in Serbia recognize the importance of winter crops like wheat and barley, as they are more stable compared to summer crops like maize and soybean, favoring their production in more drought-prone regions, especially in Northern Serbia. Apart from the more frequent drought and heat episodes, other adverse weather conditions, such as late frost and uneven spatial and temporal distribution of precipitations, limit wheat production in the Pannonian environmental zone [3].

The growth of the human population and issues of food security create major demands for sufficient and stable wheat production. Since there are limits to the further expansion of wheat cropping areas, it is necessary to increase wheat productivity [4]. Additionally, there is growing concern about the adverse effects of global warming on crop production. Heat stress has had a significant effect on about a half of the global wheat production, while the wheat cropping area affected by heat extremes is expected to expand by 2030 [5,6]. Furthermore, it is estimated that global warming will cause a decline in wheat production by about 6% for each degree Celsius rise [7]. Increasing crop yield and yield stability, as a way to respond to climate change and the rising worldwide food demand, will be a major challenge due to more frequent drought stresses in drylands, caused by global warming [8]. Water stress disturbs plant metabolic activities pressure in dryland farming while significantly reducing crop production [9]. Different cultivation practices, such as applying a combination of compost and other fertilizers, can ensure higher crop yields while also reducing the negative impact of fertilization on the environment and alleviating the effects of drought stress [10]. In addition, winter wheat production in the Serbian part of the Pannonian environmental zone will be further constrained by more frequent weather extremes, mainly heat and drought episodes [11].

Wheat is particularly susceptible to the effects of adverse weather conditions, such as temperature extremes and drought periods [12,13]. The optimal temperature for wheat anthesis is around 23 °C, while, during grain filling, it should be between 20 °C and 22 °C [14]. The effect of heat stress induces changes in physiological and molecular processes, including changes in photosynthesis, accumulation of lipids, and transcript expression, which further impacts reproductive traits and yield determination [15,16]. Heat stress commonly induces oxidative stress in wheat, which is attributed to the generation of reactive oxygen species (ROS) that can cause cellular damage [17]. Temperatures above 20 °C between spike initiation and anthesis speed up the development of spikes but reduce the number of spikelets and grains per spike [13]. At anthesis, heat stress negatively affects spike fertility by decreasing the grain number per spike [18], and, shortly before anthesis, it may have an adverse influence on floret development and potential grain weight [19]. Furthermore, high temperatures generally accelerate the rate of grain filling but shorten its duration [20]. During grain filling, heat stress promotes canopy senescence and shortens grain filling, resulting in reduced grain weight and low yield [21]. Despite these insights, past research often lacked comprehensive studies on heat stress, specifically in the Pannonian Plain, where winter wheat frequently experiences extreme temperatures (>30 °C) during critical growth phases, leading to substantial yield decline. Many studies focused on general heat stress effects, without addressing regional-specific adaptation strategies or the development of heat-resistant varieties tailored to local conditions. Therefore, to secure high and stable wheat yields, wheat production must be adapted to the changing climate in the Pannonian environmental zone. Adjusting various crop management practices, including fertilizer application and sowing time, could be an effective option for mitigating the negative effect of heat stress under the conditions in the Pannonian Plain [22]. Given that the projection of heat stress occurrence during the growing season is unreliable, which represents a notable problem in flowering window adjustment, developing heat-adapted wheat varieties should be the most promising scenario [23]. Also, despite extensive research on heat stress in wheat, there is still a significant gap in understanding how combined heat stress during anthesis and mid-grain filling impacts wheat performance. The current research often focuses on individual stress events, but due to climate change, multiple stress periods will appear more frequently. To address these gaps, a detailed analysis of how thermal stress affects the performance of different wheat varieties is necessary. Additionally, identifying potentially heat-tolerant genotypes and proposing targeted adaptation strategies would provide valuable knowledge that can be applied both locally and in broader international contexts facing similar challenges.

Heat adaptation in wheat is related to different crop physiological factors. The application of fast, low-cost, and non-destructive measurements of crop physiological traits could be a useful strategy for the selection of heat- and drought-tolerant wheat genotypes. High temperatures affect important physiological and biochemical processes of wheat plants, which could easily be assessed by handheld and non-invasive instruments [24]. Canopy and leaf temperatures are widely accepted as physiological indicators of drought and heat tolerance [25]. Under the heat stress conditions, a lower canopy temperature affects crop grain yield by stomatal opening, higher transpiration rates, increased root weight, and better water extraction [26,27]. A chlorophyll fluorescence parameter, maximum quantum efficiency of photosystem II photochemistry (Fv/Fm), shows the maximum quantum efficiency of the photosystem II (PSII) in dark-adapted leaves [28]. Measurement of chlorophyll fluorescence has been frequently used as an efficient indicator of heat stress effect on the electron transport system activity in wheat [29]. Moreover, heat stress has an adverse influence on the chlorophyll content [30]. The decrease in chlorophyll is followed by a reduced photosynthetic activity, resulting in lower grain weight and yield reduction [14]. Therefore, evaluating different physiological measurements of leaf temperature, chlorophyll fluorescence, and chlorophyll content and their relationship with the main agronomic traits could confirm the benefits of their application in wheat genotype selection and screening under different heat stress treatments.

The results presented in this study were built upon our earlier research published by Mirosavljevic et al. [16]. The current study extends the initial investigation by including additional varieties and exploring additional grain yield and physiological traits. This expansion aims to further investigate genotypic variability, with a primary focus on various physiological traits, thereby providing a more comprehensive understanding of the genetic diversity among Pannonian wheat varieties. In this study, the authors investigated changes in the physiological processes and grain yield traits in seven wheat varieties from Serbia, one from Australia, and one from the UK after seven days of exposure to high temperatures during anthesis, mid-grain filling, and their combination. Specifically, the aim was to test the hypothesis that high temperatures at different phenological stages have various negative effects on crop performance of different wheat genotypes and to assess the differences in performance among the analyzed wheat varieties. Therefore, the main goals of this study were to determine the response of the wheat varieties developed in the Pannonian environmental zone to the heat stress, as well as the difference in varietal response to heat stress at anthesis and grain filling, and to combined heat stress at both stages. Moreover, while most previous research has focused on single heat stress events, this study aims to provide new insights by addressing the relatively underexplored area of combined heat stress, offering a more comprehensive understanding of its impact on wheat performance.

## 2. Results

### 2.1. Chlorophyll Index

The chlorophyll index (CI) decreased due to the influence of mid-grain filling and combined heat stress treatment (Table 1). In general, the negative effect of heat treatment was higher at mid-grain filling compared to the control and heat stress treatment at anthesis. The effect of combined heat stress was the most severe, resulting in the highest decrease in CI values. Compared to the control, CI reduction was at 44% and 51% under heat stress at mid-grain filling and combined heat stress, respectively.

Varieties NS Rani otkos and Subotičanka had the highest CI values under the control conditions, both at anthesis and mid-grain filling. Under heat stress at anthesis, NS Rani otkos, NS Javorka, and NS Mila had the highest CI values, while NS 40s exhibited the lowest values (48.7 SPAD units). The CI values in NS Obala and Gladius were the lowest under heat stress during mid-grain filling, while the combined heat stress at both stages resulted in the lowest CI values in NS Javorka and Gladius. On the other hand, Avangarda (42.2 SPAD units) had the highest level of CI units under heat stress at mid-grain filling and the combined heat stress conditions.

### 2.2. Leaf Temperature

The LT of the studied wheat varieties was higher compared to the control under heat stress treatments at anthesis and mid-grain filling (Table 2). The leaf temperature under the heat treatments at anthesis was lower than at mid-grain filling and combined heat stress treatment. The combined heat stress effect was more pronounced than the control and single heat stress treatments and thus resulted in the highest LT. Compared to the control, LT increased by 26%, 31%, and 34% under stress conditions during anthesis, mid-grain filling, and combined stress, respectively.

The differences in leaf temperatures were not pronounced in the studied varieties under the control conditions at anthesis. Only NS Rani otkos (24.5 °C) had a lower LT than the other varieties under the control conditions at mid-grain filling. There were different genotypic responses to the heat stress influence at anthesis and mid-grain filling. NS 40s had a stable and low LT under heat stress treatments at anthesis and at mid-grain filling. On the other hand, the highest LT was recorded in Paragon, NS Obala, and Gladius under heat stress treatments at anthesis, mid-grain filling, and both stages, respectively.

### 2.3. Maximum Quantum Efficiency of PSII

There were no significant differences in Fv/Fm values between the wheat plants grown under the control and under heat stress at anthesis (Table 3). The heat stress at mid-grain filling significantly decreased the Fv/Fm as compared to the control. The lowest Fv/Fm values were recorded under combined heat stress conditions. Compared to the control, heat stress decreased theFv/Fmby63% and 74% under heat stress at mid-grain filling and combined stress, respectively.

**Figure 1:**
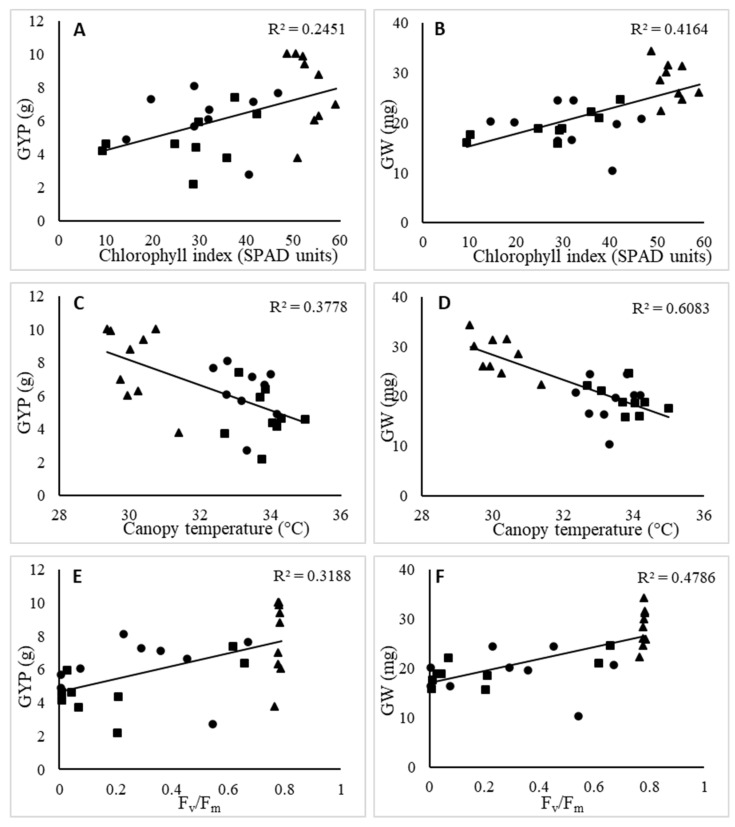
Relationship of grain yield per plant (GYP) and grain weight (GW) with chlorophyll index (**A**,**B**); leaf temperature (**C**,**D**); and maximum quantum efficiency of PSII (Fv/Fm) (**E**,**F**) of winter wheat varieties under heat stress (heat stress at anthesis: triangle, heat stress at mid-grain filling: circle, and combined heat stress at anthesis and mid-grain filling: square);The coefficients of determination were significant at the 0.01 probability level.

There were no significant differences among wheat varieties in the Fv/Fm at anthesis under the control conditions. Under heat stress at anthesis, the differences among the varieties were less pronounced, although Paragon (0.765 Fv/Fm) showed the lowest value of Fv/Fm. Avangarda had the highest Fv/Fm under heat stress at mid-grain filling and combined heat stress. On the other hand, the lowest Fv/Fm were recorded in NS Javorka and NS Obala under heat stress at mid-grain filling, while Gladius and NS Javorka had the lowest Fv/Fm under combined heat stress treatment. With respect to the maximum quantum efficiency of PSII, there was a positive association with grain yield and grain weight under the condition of increased temperature (Figure 1E,F).

### 2.4. Grain Yield, Grain Weight, and Grain Number per Plant

The GYP varied significantly across the stress treatments (Table 4). Heat stress at anthesis reduced GYP by 32% as compared to the control plants, while heat stress at mid-grain filling resulted in a 46% GYP decrease. Moreover, GYP decreased more than twice in comparison to the control treatment under combined heat stress.

The studied wheat varieties showed a notable GYP variation across different stress treatments. Under control conditions, the highest GYP was recorded in NS Mila (14.41 g), while Subotičanka (7.36 g) showed the lowest GYP. Heat stress at anthesis decreased the GYP in all varieties, where Paragon, Subotičanka, and NS Javorka were the lowest yielding varieties. At the heat stress at mid-grain filling treatment, NS Mila had a higher GYP than the other varieties, while the lowest GYP was recorded in Paragon. NS 40s (7.37 g) showed the highest GYP under the combined heat stress treatment, whereas Paragon (2.17 g) was the lowest yielding variety.

Across the treatments, GW showed a general pattern with the highest values under the control conditions and under heat stress at anthesis. It also showed a significant reduction at heat stress during mid-grain filling and combined heat stress treatment at both stages (Table 5). Both heat stress during mid-grain filling and combined heat stress approximately reduced the GW by 30%. Paragon had the lowest GW under control and all the heat stress treatments. Avangarda showed high GW under the control and combined heat stress. The highest GW under heat stress during anthesis was recorded in NS 40s (34.17 g), while NS Mila (24.44 g) and Subotičanka (24.53 g) had the highest values under the heat stress during mid-grain filling.

The NGP decreased significantly under heat treatment at anthesis and at mid-grain filling (Table 6). The negative effect of the heat treatment was more prominent at anthesis than during mid-grain filling and at the control conditions.

The influence of the combined heat stress at anthesis and mid-grain filling was the most severe, resulting in the highest decrease of NGP. Paragon (685) had the highest NGP under the control conditions, while, under heat stress, Paragon was among the varieties with the lowest value of this trait, showing the highest reduction. Gladius had the highest NGP (365) under heat stress during anthesis. The highest NGP was recorded in NS 40s under heat stress during mid-grain filling and combined heat stress at both stages.

### 2.5. Stress Index

Across treatments, the varieties NS 40s, Avangarda, Gladius, and NS Obala exhibited higher values of the harmonic mean stress tolerance index (HM) under heat stress at anthesis (H-anthesis), while Paragon had lower values compared to the other varieties (Table 7). During heat stress at mid-grain filling (H-filling), NS Mila showed the highest tolerance among the varieties, while Paragon again had the lowest tolerance. Under combined heat stress at anthesis and grain filling (H-anthesis + filling), NS 40s, Avangarda, and NS Mila showcased the highest values at 9.3, 8.7, and 8.4, respectively, with Paragon once again displaying the lowest value at 3.5.

### 2.6. Relationships between Traits

The results from this study showed variation in the relationships between the main grain yield traits (GYP and GW) and different physiological traits under heat stress conditions (Figure 1). The relationship of CI with the GYP and GW was positive under the heat stress conditions, showing a higher association with grain weight (Figure 1A,B). Under the heat stress conditions, LT was negatively related to both grain weight and grain yield per plant, showing a higher relationship with GW (Figure 1C,D). With respect to the maximum quantum efficiency of PSII, there was a positive association with grain yield and grain weight under conditions of increased temperature (Figure 1E,F).

## 3. Discussion

Despite extensive research on the impact of heat stress on wheat grain yield and physiology [31,32], the cultivation of wheat under high temperature conditions continues to represent significant challenges both for breeders and producers. To identify changes in physiological traits related to grain yield, wheat plants were subjected to high temperature stress during two main growth stages: anthesis and mid-grain filling. These stages were selected due to their critical importance in grain yield formation [33,34]. Considering the rise of multiple heat stress events in the future due to climate change [11], we extended our study beyond single heat stress treatments. In addition to subjecting the plants to heat stress during anthesis and mid-grain filling, we also applied heat stress during both stages. This approach enabled us to analyze and compare the effects of single and multiple heat stress events on various physiological and grain yield traits, providing new insights into the wheat response to complex environmental conditions.

### 3.1. Grain Weight and Grain Number

To the best of our knowledge, there has been no information on the performance of modern Pannonian wheat varieties under multiple heat stress conditions that could ensure sustainable crop production under the current climate conditions and warmer climate conditions predicted in the future. Our observation of decreased grain yield traits under heat stress aligns with previous studies that have documented the negative impact of high temperatures on wheat productivity [35,36]. However, the responses of the analyzed traits varied across different heat stress treatments (Table 4, Table 5 and Table 6). While the combined heat stress had the most severe effects, the effect of heat stress on some traits at mid-grain filling was more adverse than at anthesis and vice versa. Consistent with the earlier research, the negative effect of heat stress at anthesis had a more severe effect on NGP, while heat stress during mid-grain filling resulted in the greatest GW decline [37]. Generally, the decline of GW was related to changes in the grain filling traits [38], while the NGP was a result of flower abortion and pollen sterility [39]. Additionally, the decrease in GW under high temperature stress conditions was found to result from the reduction in leaf chlorophyll content and maximum quantum yield [40], as well as the diminished staygreen ability [41]. An increase in the maximum temperature around mid-anthesis, particularly one day after 50% anthesis, can lead to a 40% reduction in the number of grains per year, significantly decreasing grain yield [42].

### 3.2. Grain Yield

Concerning GYP, the effect of the heat stress during mid-grain filling was more severe than during anthesis, while the combined heat stress treatment showed synergistic detrimental effects on GYP, due to the adverse effect on both NGP and GW. Among the modern wheat varieties, Avangarda performed well under heat stress conditions and constantly ranked high among the most yielding varieties (Table 4). Additionally, it exhibited high values of the stress index, indicating its superior heat stress tolerance compared to the other varieties. These results suggest that Avangarda is suitable for environments characterized by heat stress occurrence during anthesis and the mid grain-filling period. NS 40s was able to maintain a high yield under heat stress at anthesis and combined heat stress treatment at both stages, while the previously characterized heat-tolerant variety Gladius had a high grain yield under heat stress at anthesis and mid-grain filling. Agronomically, the GYP of variety Paragon was based on the higher GNP production and low grain weight under the control conditions. However, heat stress during different stages notably reduced the GNP in Paragon, resulting in a remarkable GYP reduction and low heat stress index that could not be compensated for with higher grain weight stability. Despite being exposed to combined stresses during both anthesis and grain filling, resulting in a more severe GYP reduction compared to single stress in most varieties, this phenomenon was not observed in NS 40s. Contrary to expectations, the NS 40s variety had a greater reduction in GYP under stress conditions at grain filling compared to the combined stress. The heat stress during anthesis may have triggered some specific physiological adjustments in the NS 40s variety, such as altered hormone signaling pathways or changes in gene expression related to stress tolerance, potentially resulting in a less pronounced GYP loss. Significant genotypic variation was observed in the response of wheat varieties to heat stress during anthesis and grain filling in different studies [34,43]. Understanding and exploiting the natural genetic variation in physiological, reproductive, and quality traits can accelerate breeding for heat tolerance through conventional and innovative approaches [44].

### 3.3. Chlorophyll Flourescence

The Fv/Fm parameter can be used as an efficient screening tool for heat stress-tolerant genotypes under controlled and field conditions [45]. The primary photochemical reactions measured as a maximum quantum yield of PSII photochemistry showed a significant decrease under heat stress during grain filling, while the negative effect of heat stress at anthesis was not recorded, showing a decreased value only in Paragon (Table 3). Theabsence of a high temperature effect at anthesis was also found among the Czech wheat varieties [46], indicating that temperatures of 35/25 °C day/night for a seven-day period are not adequate for the differentiation of wheat variety reactions according to Fv/Fm measurements. By increasing the temperatures to 38/28 °C during mid-grain filling and combined stress, a significant diversity in Fv/Fm values was recorded among the studied wheat varieties. The variety Avangarda showed the highest value of Fv/Fm under heat stress at mid-grain filling and combined stress, maintaining a better photosynthetic performance compared to the other varieties. The combined influence of heat stress at anthesis and mid-grain filling amplified the Fv/Fm decrease in wheat varieties compared to the control and single heat stress treatments at either the anthesis or mid-grain filling stages. This reduction could be related to the previous significant chlorophyll index decrease at anthesis, since temperatures below 38 °C during anthesis do not have a significant influence on the Fv/Fm values in the wheat varieties [29]. We found that higher Fv/Fm values of wheat varieties during heat stress were positively related to both GYP and GW (Figure 1), while the relationship with NGP was not significant. Maintaining photosynthesis during this critical stage is crucial, as there is a significant positive correlation of the photosynthetic rate with GW and GYP [37]. Our findings regarding the close correlation between Fv/Fm and the yield traits align with the previous research, indicating the application of Fv/Fm as an indicator of plant physiological stress and its implications for yield performance [47]. Heat-tolerant wheat lines maintain higher values of key chlorophyll fluorescence parameters like Fv/Fm (maximum quantum efficiency of PSII) under heat stress compared to susceptible lines, as previously reported [29].

### 3.4. Chlorophyll Index

The average reduction of CI was similar to the Fv/Fm response, since wheat varieties showed a lower decrease (6%) under heat stress during anthesis, while CI reduction under heat stress at mid-grain filling and combined heat stress at both stages was more pronounced (Table 1). The decrease in chlorophyll content in leaves could be related to the inhibition of chlorophyll synthesis [48], thylakoid membrane damage [49], and membrane lipid peroxidation [50]. Although heat-induced accelerated senescence during mid-grain filling is already recognized as the crop mechanism for survival [51], there was a significant relationship of CI with grain yield per plant and grain weight, indicating that the crop ability to maintain higher CI values under heat stress could be used as a valuable criterion in the selection of heat-tolerant genotypes. The results also showed a significant variation in CI under heat stress, especially during mid-grain filling and combined heat stress. Our findings regarding the different responses of wheat varieties to heat stress are consistent with the previous results, indicating that genetic variation plays a crucial role in determining heat tolerance [44]. With respect to the Fv/Fm, Avangarda had the highest CI values under heat stress at mid-grain filling and combined heat stress, maintaining a higher CI during the heat treatments compared to the other varieties. On the other hand, mid-grain filling and combined heat stress accelerated leaf senescence and higher chlorophyll loss, mostly in NS Obala and Paragon, respectively. Similarly, previous studies compared heat-tolerant and heat-sensitive wheat varieties and demonstrated that heat stress significantly reduced the chlorophyll content in all varieties, albeit to a lesser extent in the tolerant lines [52].

### 3.5. Leaf Temperature

We found a significant genotypic variation in LT, which was already reported for the controlled and field conditions [53]. Wheat genotypes vary in their ability to maintain leaf temperatures under heat stress, with some genotypes demonstrating greater heat tolerance by maintaining lower canopy temperatures, thus reflecting better physiological adaptation to high temperatures [36]. The varieties that experienced lower yield reductions under heat stress, such as NS 40s and Avangarda, tend to retain lower LT compared to the other varieties under the same growing conditions (Table 2). This observation aligns with previous research findings by Kaur et al. [54], who reported that wheat genotypes that maintain lower canopy temperatures under heat stress tend to have higher grain yields compared to heat-sensitive genotypes. Nevertheless, the variety Paragon performed poorly regarding GYP under heat stress at the mid-grain filling and combined heat stress treatments and did not show a notably higher CT compared to the other varieties. Accordingly, the LT threshold for heat tolerance may depend on the variety and the time of measurements. Under heat conditions, wheat varieties with cooler leaf temperatures probably achieved higher stomatal conductance and net photosynthesis rates, resulting in increased GW and GYP [36,37,40,41,42,43,44,45,46,47,48,49,50,51,52,53,54,55].

## 4. Materials and Methods

### 4.1. Plant Materials

In this study, nine wheat varieties were utilized, of which seven originated from Serbia, one from Australia, and one from the UK (Table 8). The selection of Serbian wheat varieties aimed to represent modern varieties, widely grown and well-adapted to the Pannonian conditions. Considering a significant genotypic variation within wheat varieties, the most representative varieties were selected to efficiently manage different combinations of stress treatments during the experiment. Varieties Gladius from Australia, adapted to more heat-prone climates, and Paragon from the United Kingdom, adapted to a cool and temperate climate, were selected as check varieties according to the previously determined difference in stress tolerance [56]. Although nine wheat genotypes were included in the experiment, the difference in the anthesis date between the earliest and the latest genotypes was approximately five days. Therefore, minor phenological differences were not a major factor in genotype responses to high temperature. The experiment was conducted in a phenotyping platform under a controlled environment at the Department of Food Science, Aarhus University, Denmark.

In each pot (measuring 9 cm in height and 11 cm in diameter), which contained a commercial peat-based potting substrate composed of sphagnum peat with added clay, NPK fertilizer, micronutrients, and superphosphate (Pindstrup Færdigblanding 2, produced by Pindstrup Mosebrug A/S, Ryomgaard, Denmark; https://www.pindstrup.dk/professionel/product-details/pindstrup-f%C3%A6rdigblanding-2, accessed on 24 June 2024), two seeds of the selected wheat genotypes were planted. The plants were cultivated under greenhouse conditions with a 12-h day/night photoperiod, utilizing both natural and supplementary light sources at the Department of Food Science, Aarhus University, Aarslev, DK (55°18′27″ N 10°26′35″ E). The relative air humidity was maintained at 48 ± 5%, with an average temperature of 23.8 ± 1.5 °C and ambient CO_2_ levels until the three-leaf stage. High-pressure sodium lamps (SON-T Agro, 600 W, Philips, Eindhoven, The Netherlands) were used to supplement lighting whenever the natural photosynthetic photon flux density (PPFD) dropped below 150 µmol m^−2^ s^−1^.Subsequently, the pots were thinned to one plant per plot and transferred to a cold chamber for a six-week vernalization period at 4–6 °C, with 8 h of daylight. After vernalization, the plants were reintroduced to the greenhouse and grown until anthesis (Zadoks 60). At the onset of anthesis (Zadoks 61), 20 plants of each variety were acclimatized for two days in climate chambers (MB Teknik, Brøndby, Denmark). The climate chamber settings were maintained at 24/16 °C (14/10 h day/night), with a PPFD of 500 μmol m^−2^ s^−1^ (LED FL300 Sunlight, Fionia Lighting, Søndersø, Denmark), a relative air humidity of 65%, and CO_2_ levels of 400 ppm.

The responses of wheat plants to heat stress were evaluated under fully controlled environmental conditions in separate chambers. Control plants were maintained at 24/16 °C for 14/10 h day/night. Heat stress during anthesis (T2–Zadoks 65) was induced at 35/25 °C for seven days, while heat stress during mid-grain filling (T3) was applied at 38/28 °C for the same duration. Combined heat stress (T4) involved treatments at 35/25 °C during anthesis and at 38/28 °C during mid-grain filling (Zadoks 70–75), both for seven days under a 14/10-h day/night period. The plants received irrigation by flooding the bench with a nutrient solution (190 ppm N, 35 ppm P, and 275 ppm K, pH 6.0) for approximately 10 min three times per day. Following the heat stress treatments, the plants were returned to the greenhouse and cultivated under the previously described conditions.

### 4.2. Measurements

The chlorophyll index (CI) of the flag leaf was measured in vivo, using a handheld chlorophyll meter SPAD-502 (Minolta Ltd., Osaka, Japan), at three random spots on the upper leaf surface of five plants per treatment on the seventh day of stress. For monitoring the maximum quantum efficiency of PSII photochemistry (Fv/Fm), five plants per variety per treatment were assessed using a Mini-Pam fluorimeter (Walz Gmbh, Effeltrich, Germany) seven days after exposure to the stress conditions. The plants were dark-adapted for at least 30 min using dark clips, and Fv/Fm was measured on the upper leaf surface using the photosynthetic photon flux density of 3500 μmol m^−2^ s^−1^ as a saturating flash. Leaf temperature (LT) of five plants per variety was recorded using a Raynger 3i infrared gun (Raytek, Santa Cruz, CA, USA). Measurements were taken at two randomly selected spots on the main flag leaf during the 10th hour of light exposure on the seventh stress day. The plants were cultivated in the greenhouse until reaching the full ripening stage (Zadoks’ growth stage 92); at which point, they were manually harvested. Individual plant spikes were harvested from each pot. Grain yield-related traits, including grain yield per plant (GYP) and number of grains per plant (NGP), were determined after manual threshing, while grain weight (GW) was calculated as the ratio between GYP and NGP.

### 4.3. Statistical Analysis

The analysis of variance (ANOVA) for all measured parameters was conducted using Infostat software (version 2014). Tukey’s test (*p* < 0.05) was employed to assess the significance among different treatments, as well as among the varieties within each treatment and at each time of measurement. The following calculations were used to compute the harmonic mean heat tolerance index: Harmonic mean (HM) = 2(Yp × Ys)/(Yp + Ys), where Yp and Ys are the yield performance of the varieties [57]. Linear regression analysis was utilized to investigate the relationship between various physiological traits measured on the seventh day of stress and grain yield, as well as grain weight, under heat conditions.

## 5. Conclusions

In conclusion, our results showed that the effect of combined heat stress at both stages was more severe than each single heat stress, indicating that the interaction between multiple heat stress treatments was synergistic. The findings emphasize the importance of selecting heat-tolerant wheat varieties and the potential for using the chlorophyll index, leaf temperature, and the maximum quantum efficiency of PSII as effective screening tools for heat tolerance in wheat breeding programs. Among the tested modern Pannonian wheat varieties, there is a notable variation in the heat stress response, enabling the identification of wheat varieties, such as Avangarda and NS 40s, as a heat-tolerant material. These varieties should be widely included in further breeding activities under the conditions in the Pannonian Plain as valuable sources of heat stress tolerance.

## Figures and Tables

**Table 1:** Chlorophyll index (SPAD units) of nine wheat varieties at the seventh day under control conditions at anthesis (C-anthesis), heat stress at anthesis (H-anthesis), control conditions at mid-grain filling (C-filling), heat stress at mid-grain filling (H-filling), and combined heat stress at anthesis and mid-grain filling (H-anthesis + filling).

Varieties	Treatments				
	C-Anthesis	H-Anthesis	C-Filling	H-Filling	H-Anthesis + Filling
NS 40s	51.7^c^ ± 1.2	48.7^d^ ± 2.5	51.8^d^ ± 1.5	31.9^cd^ ± 4.8	37.6^ab^ ± 4.2
Avangarda	53.4^c^ ± 1.7	52.0^bcd^ ± 1.3	54.6^bcd^ ± 0.8	46.7^a^ ± 3.7	42.2^a^ ± 9.5
Gladius	55.4^bc^ ± 0.6	50.6^cd^ ± 1.2	54.5^bcd^ ± 1.1	19.6^ef^ ± 3.6	10.1^d^ ± 6.4
NS Javorka	60.2^a^ ± 2.9	55.4^ab^ ± 1.3	56.7^b^ ± 2.1	28.8^de^ ± 2.5	9.4^d^ ± 4.9
NS Mila	58.9^ab^ ± 2.2	55.5^ab^ ± 2.6	55.6^bc^ ± 2.4	28.8^de^ ± 3.5	29.8^bc^ ± 0.28
NS Obala	54.2^c^ ± 2.0	52.4^bcd^ ± 1.9	54.3^bcd^ ± 2.3	14.4^f^ ± 6.8	24.7^c^ ± 2.8
Paragon	52.5^c^ ± 2.5	50.9^cd^ ± 2.4	52.7^cd^ ± 2.1	40.5^abc^ ± 3.7	28.8^bc^ ± 1.5
NS Rani otkos	61.3^a^ ± 2.4	59.1^a^ ± 1.6	62.7^a^ ± 1.1	41.4^ab^ ± 6.1	29.2^bc^ ± 5.8
Subotičanka	61.2^a^ ± 1.5	54.5^bc^ ± 2.8	60.5^a^ ± 1.9	32.1^bcd^ ± 4.1	35.9^ab^ ± 5.8
average	56.5A ± 4.1	53.2B ± 3.5	55.9A ± 3.7	31.6C ± 10.7	27.5D ± 11.9

Different lowercase letters indicate statistically significant differences among variety means within a treatment tested by one-way ANOVA with Tukey’s post hoc test (*p* < 0.05). Different uppercase letters indicate statistically significant differences among treatment averages tested by one-way ANOVA with Tukey’s post hoc test (*p* < 0.05).Mean ± standard deviation of five replicates.

**Table 2:** Leaf temperature (°C) of nine wheat varieties on the seventh day under control conditions at anthesis (C-anthesis), heat stress at anthesis (H-anthesis), control conditions at mid-grain filling (C-filling), heat stress at mid-grain filling (H-filling), and combined heat stress at anthesis and mid-grain filling (H-anthesis + filling).

Varieties	Treatments				
	C-Anthesis	H-Anthesis	C-Filling	H-Filling	H-Anthesis + Filling
NS 40s	24.2^a^ ± 0.20	29.4^d^ ± 0.33	25.2^ab^ ± 0.13	32.7^cd^ ± 0.11	33.1^cd^ ± 0.14
Avangarda	23.7^a^ ± 0.36	29.5^cd^ ± 0.23	25.3^a^ ± 0.19	32.4^d^ ± 0.22	33.7^bc^ ± 1.09
Gladius	24.2^a^ ± 0.58	30.7^ab^ ± 1.00	25.6^a^ ± 0.43	34.0^ab^ ± 0.12	35.0^a^ ± 0.19
NS Javorka	24.0^a^ ± 0.81	30.3b^cd^ ± 0.52	25.4^a^ ± 0.43	33.2^bcd^ ± 0.17	34.2^ab^ ± 028
NS Mila	23.5^a^ ± 0.36	30.0^bcd^ ± 0.13	25.6^a^ ± 054	32.8^cd^ ± 0.25	33.7^bcd^ ± 0.37
NS Obala	24.0^a^ ± 0.47	30.4^bc^ ± 0.20	25.1^ab^ ± 0.33	34.2^a^ ± 0.46	34.3^ab^ ± 0.40
Paragon	24.4^a^ ± 0.47	31.4^a^ ± 0.54	25.1^ab^ ± 0.13	33.3^bc^ ± 0.58	33.8^bc^ ± 0.44
NS Rani otkos	23.5^a^ ± 0.40	29.7^cd^ ± 0.28	24.5^b^ ± 0.22	33.5^abc^ ± 083	34.1^abc^ ± 0.51
Subotičanka	23.9^a^ ± 0.44	29.9^bcd^ ± 0.27	25.5^a^ ± 0.42	33.8^ab^ ± 0.35	32.7^d^ ± 0.25
average	23.9E ± 0.53	30.1C ± 0.74	25.3D ± 0.47	33.3B ± 0.7	33.9A ± 0.78

Different lowercase letters indicate statistically significant differences among variety means within a treatment tested by one-way ANOVA with Tukey’s post hoc test (*p* < 0.05). Different uppercase letters indicate statistically significant differences among treatment averages tested by one-way ANOVA with Tukey’s post hoc test (*p*< 0.05).Mean ± standard deviation of five replicates.

**Table 3:** Maximum quantum efficiency of PSII (Fv/Fm) of nine wheat varieties on the seventh day under control conditions at anthesis (C-anthesis), heat stress at anthesis (H-anthesis), control conditions at mid-grain filling (C-filling), heat stress at mid-grain filling (H-filling), and combined heat stress at anthesis and mid-grain filling (H-anthesis + filling).

Varieties	Treatments				
	C-Anthesis	H-Anthesis	C-Filling	H-Filling	H-Anthesis + Filling
NS 40s	0.805^a^ ± 0.010	0.782^ab^ ± 0.007	0.794^ab^ ± 0.003	0.075^ef^ ± 0.109	0.618^a^ ± 0.109
Avangarda	0.804^a^ ± 0.005	0.782^ab^ ± 0.003	0.793^ab^ ± 0.009	0.670^a^ ± 0.095	0.659^a^ ± 0.076
Gladius	0.801^a^ ± 0.008	0.779^ab^ ± 0.009	0.787^b^ ± 0.012	0.290^cd^ ± 0.159	0.011^d^ ±0.009
NS Javorka	0.808^a^ ± 0.003	0.779^ab^ ± 0.004	0.761^c^ ± 0.013	0.005^f^ ± 0.003	0.010^d^ ± 0.005
NS Mila	0.810^a^ ± 0.004	0.784^ab^ ± 0.010	0.803^ab^ ± 0.05	0.228^de^ ± 0.018	0.029^cd^ ± 0.023
NS Obala	0.804^a^ ± 0.012	0.785^a^ ± 0.009	0.797^ab^ ± 0.007	0.004^f^ ± 0.006	0.045^bcd^ ± 0.049
Paragon	0.806^a^ ± 0.006	0.765^b^ ± 0.017	0.805^a^ ± 0.002	0.543^ab^ ± 0.121	0.206^bc^ ± 0.150
NS Rani otkos	0.808^a^ ± 0.005	0.779^ab^ ± 0.015	0.800^ab^ ± 0.006	0.358^bcd^ ± 0.105	0.211^b^ ± 0.134
Subotičanka	0.811^a^ ± 0.003	0.788^a^ ± 0.003	0.804^a^ ± 0.003	0.453^bc^ ± 0.106	0.069^bcd^ ± 0.068
average	0.806A ± 0.007	0.780A ± 0.011	0.794A ± 0.015	0.291B ± 0.243	0.206C ± 0.257

Different lowercase letters indicate statistically significant differences among variety means within a treatment tested by one-way ANOVA with Tukey’s post hoc test (*p*< 0.05). Different uppercase letters indicate statistically significant differences among treatment averages tested by one-way ANOVA with Tukey’s post hoc test (*p*< 0.05).Mean ± standard deviation of five replicates.

**Table 4:** Grain yield per plant (GYP; g) of nine wheat varieties grown under control conditions, and heat treatments at anthesis (H-anthesis), at mid-grain filling (H-filling), and combined heat stress at anthesis and mid-grain filling (H-anthesis + filling).

Varieties	Treatments			
	C-Anthesis	H-Anthesis	H-Filling	H-Anthesis + Filling
NS 40s	12.68^abc^ ± 0.88	9.99^a^ ± 1.02	6.08^bcd^ ± 0.62	7.37^a^ ± 0.57
Avangarda	13.77^ab^ ± 0.90	9.88^a^ ± 1.36	7.68^ab^ ± 0.62	6.37^a^ ± 0.78
Gladius	11.79^bcd^ ± 0.86	10.02^a^ ± 1.16	7.29^abc^ ± 0.97	4.58b^c^ ± 1.55
NS Javorka	11.33^cd^ ± 0.68	6.29^c^ ± 0.45	5.70^cd^ ± 1.48	4.15^c^ ± 0.63
NS Mila	14.41^a^ ± 1.34	8.77^ab^ ± 1.07	8.12^a^ ± 0.56	5.92^ab^ ± 0.69
NS Obala	11.76^bcd^ ± 0.76	9.38^a^ ± 1.21	4.90^d^ ± 0.66	4.60^bc^ ± 0.40
Paragon	10.20^d^ ± 1.23	3.76^d^ ± 1.00	2.75^e^ ± 0.54	2.17^d^ ± 0.55
NS Rani otkos	11.88^bcd^ ± 1.29	6.98^bc^ ± 0.60	7.14^abc^ ± 1.09	4.36^bc^ ± 0.80
Subotičanka	7.36^e^ ± 0.60	6.03^c^ ± 0.45	6.65^abc^ ± 0.38	3.72^cd^ ± 056
average	11.69A ± 2.05	7.90B ± 2.30	6.26C ± 1.75	4.80D ± 1.64

Different lowercase letters indicate statistically significant differences among variety means within a treatment tested by one-way ANOVA with Tukey’s post hoc test (*p*< 0.05). Different uppercase letters indicate statistically significant differences among treatment averages tested by one-way ANOVA with Tukey’s post hoc test (*p*< 0.05).Mean ± standard deviation of five replicates.

**Table 5:** Grain weight (GW; g) of nine wheat varieties grown under control conditions, and heat treatments at anthesis (H-anthesis), at mid-grain filling (H-filling), and combined heat stress at anthesis and mid-grain filling (H-anthesis + filling).

Varieties	Treatments			
	C-Anthesis	H-Anthesis	H-Filling	H-Anthesis + Filling
NS 40s	26.7^b^ ± 1.04	34.17^a^ ± 2.05	16.49^c^ ± 2.99	20.96^abc^ ± 1.30
Avangarda	31.36^a^ ± 2.39	29.95^abc^ ± 3.82	20.77^ab^ ± 0.55	24.59^a^ ± 1.07
Gladius	29.85^ab^ ± 1.34	28.38^abcd^ ± 6.19	20.17^bc^ ± 1.98	17.56^cd^ ± 2.03
NS Javorka	26.40^b^ ± 1.47	24.49^cd^ ± 2.03	16.39^c^ ± 1.22	15.89^d^ ± 1.39
NS Mila	28.83^ab^ ± 2.69	31.27^abc^ ± 1.53	24.44^a^ ± 2.67	18.79^bcd^ ± 1.52
NS Obala	31.75^a^ ± 1.75	31.46^ab^ ± 3.22	20.24^bc^ ± 2.15	18.79^bcd^ ± 2.06
Paragon	14.85^c^ ± 1.17	22.29^d^ ± 4.29	10.36^d^ ± 1.73	15.71^d^ ± 2.73
NS Rani otkos	27.00^b^ ± 1.20	26.02^bcd^ ± 1.45	19.72^bc^ ± 1.19	18.48^bcd^ ± 3.44
Subotičanka	27.67^b^ ± 1.38	25.88^bcd^ ± 1.37	24.53^a^ ± 0.70	22.15^ab^ ± 1.49
average	27.16A ± 5.00	28.21A ± 4.7	19.23B ± 4.51	19.21B ± 3.32

Different lowercase letters indicate statistically significant differences among variety means within a treatment tested by one-way ANOVA with Tukey’s post hoc test (*p* < 0.05). Different uppercase letters indicate statistically significant differences among treatment averages tested by one-way ANOVA with Tukey’s post hoc test (*p* < 0.05).Mean ± standard deviation of five replicates.

**Table 6:** Number of grains per plant (NGP) of nine wheat varieties grown under the control conditions, and heat treatments at anthesis (H-anthesis), at mid-grain filling (H-filling), and combined heat stress at anthesis and mid-grain filling (H-anthesis + filling).

Varieties	Treatments			
	C-Anthesis	H-Anthesis	H-Filling	H-Anthesis + Filling
NS 40s	475^b^ ± 27	293^abc^ ± 33	378^a^ ± 80	352^a^ ± 20
Avangarda	441^bc^ ± 38	332^ab^ ± 47	369^ab^ ± 26	259^bc^ ± 30
Gladius	395^c^ ± 25	365^a^ ± 76	362^abc^ ± 37	257^bc^ ± 63
NS Javorka	430^bc^ ± 23	258^bcd^ ± 20	346^abc^ ± 78	262^ab^ ± 48
NS Mila	501^b^ ± 33	281^abc^ ± 37	337^abcd^ ± 55	314^ab^ ± 21
NS Obala	372^c^ ± 25	302^abc^ ± 62	243^d^ ± 28	246^bc^ ± 28
Paragon	685^a^ ± 60	167^d^ ± 27	265^cd^ ± 12	142^d^ ± 48
NS Rani otkos	441^bc^ ± 54	269^bc^ ± 36	363^abc^ ± 57	243^bc^ ± 68
Subotičanka	266^d^ ± 24	234^cd^ ± 30	271^bcd^ ± 10	169^cd^ ± 33
average	445A ± 112	278C ± 67	326B ± 67	225D ± 73

Different lowercase letters indicate statistically significant differences among variety means within a treatment tested by one-way ANOVA with Tukey’s post hoc test (*p* < 0.05). Different uppercase letters indicate statistically significant differences among treatment averages tested by one-way ANOVA with Tukey’s post hoc test (*p* < 0.05).Mean ± standard deviation of five replicates.

**Table 7:** Harmonic mean stress tolerance index of nine wheat varieties grown under the control conditions, and heat treatments at anthesis (H-anthesis), at mid-grain filling (H-filling), and combined heat stress at anthesis and mid-grain filling (H-anthesis + filling).

Varieties	Treatments		
	H-Anthesis	H-Filling	H-Anthesis + Filling
NS 40s	30.0^a^ ± 3.07	8.2^cd^ ± 0.62	9.3^a^ ± 0.67
Avangarda	29.6^a^ ± 4.08	9.8^ab^ ± 0.64	8.7^a^ ± 0.74
Gladius	30.1^a^ ± 3.49	9.0^abc^ ± 0.82	6.5^b^ ± 1.62
NS Javorka	18.9^c^ ± 1.36	7.6^cd^ ± 1.31	6.0^b^ ± 0.63
NS Mila	26.3^ab^ ± 3.20	10.4^a^ ± 0.46	8.4^a^ ± 0.90
NS Obala	28.1^a^ ± 3.63	6.9^d^ ± 0.76	6.6^b^ ± 0.49
Paragon	11.3^d^ ± 2.99	4.3^e^ ± 0.57	3.5^c^ ± 0.61
NS Rani otkos	20.9^bc^ ± 1.79	8.8^bc^ ± 0.48	6.3^b^ ± 0.74
Subotičanka	18.1^c^ ± 1.35	7.0^d^ ± 0.39	4.9^bc^ ± 0.40

Different letters indicate statistically significant differences among treatment means tested by one-way ANOVA with Tukey’s post hoc test (*p* < 0.05). Mean ± standard deviation of five replicates.

**Table 8:** Varieties used to assess the heat stress effects on physiological parameters and agronomic traits.

Variety Name	Country of Origin	Anthesis Date	Response to Heat Stress
NS 40s	Serbia	Medium late	Unknown
Avangarda	Serbia	Medium late	Unknown
NS Javorka	Serbia	Medium early	Unknown
NS Mila	Serbia	Medium late	Unknown
NS Rani otkos	Serbia	Early	Unknown
NS Obala	Serbia	Medium late	Unknown
Subotičanka	Serbia	Medium late	Unknown
Paragon	United Kingdom	Medium early	Sensitive
Gladius	Australia	Medium late	Tolerant

## Data Availability

The datasets generated for this study are available on request to the corresponding author.
